# Women as Doctors

**Published:** 1873-12

**Authors:** 


WOMEN AS DOCTORS.—*Miss Sophia Jex-Blake* writes to the *Lancet:*—"You remark that 'none of the female aspirants to medical practice have ever been known to hesitate to practice the art of medicine and surgery on men.' Had my paper at Norwich been more fully reported, you would have found in it these words: 'It is for the medical practice of women among their own sex, *and for this alone,* that I plead.' So far as I am aware, none of the four ladies now practicing in England are in the habit of attending male patients; nor have I, nor, as I believe, any of my fellow-students at Edinburgh, any such idea."

